# A Note on a Case of Traumatic Encephalocele

**Published:** 1910-04-16

**Authors:** 


### Surgery.

### A NOTE ON A CASE OF TRAUMATIC ENCEPHALOCELE.

Any genuine case of traumatic encephalocele is sufficiently rare to excite remark, but there are points in the case, the history of which is about to be recorded, which are of unusual interest.

The patient, a lad aged twenty years, sought advice on account of severe and constant pain in the head, more or less localised to the seat of an old injury. It appeared that his condition after the original accident excited some interest, and he was apparently shown at a medical society, for he brought with him a copy of the notes which were then shown, with a transcript of the comments which were made by the medical men who saw him.

### History of the Case.

The history was as follows: When he was six months old he was being carried by his sister when he was dropped, the right side of his head striking the ground. He was immediately rendered unconscious, and remained so for ten days. A hæmatoma was found in the region of the right parietal eminence, but no mention was made of pulsation being subsequently discovered in it, nor of there being any definite evidence of the presence of a simple or depressed fracture of the skull. An operation was advised, but permission was absolutely refused by the parents; and the child was accordingly kept in bed and the head was packed in ice for three weeks. This was described at the medical meeting referred to as "a treatment sufficient to kill any ordinary child"—a not entirely unjustifiable criticism. The child, however, recovered consciousness and survived. During convalescence it was discovered that he had a complete left-sided hemiplegia, there being left facial paralysis and complete loss of power in left arm and left leg. The limbs recovered gradually, but the facial weakness persisted, and this was the only permanent ill-effect of the injury. His growth was not interfered with, nor his intellectual power; in fact, he reached the top standard at school at an earlier age than the majority of boys do. But as he grew up he noticed "a throbbing swelling" on the right side of the head, and for this reason he particularly disliked having his hair cut by a barber, a dislike which was accentuated by the fact that one of them once made some joking remark about it.

Ten days before he came under observation by the writer he had begun to notice pain at the site of the old injury, which had been constant. He said that sometimes he felt as if a wedge were being driven into his head, and at others as if his head were going to burst. At the same time the edges of the throbbing swelling became excessively tender, so that he could not bear anyone to touch them. He had for the same length of time felt giddy, and he had been unable to sleep. He had also gradually increasing loss of power in the left arm and hand.

When seen the only obvious abnormality was a left facial paralysis of the upper neuron type. The grip of the left hand was distinctly weak, but the left leg was normal. There was no anæsthesia. On examining the skull an oblong pulsating swelling was discovered, two and a half inches long, in the antero-posterior direction, and three-quarters of an inch wide, but its exact contour was difficult to make out on account of the extreme tenderness. In fact, this was so marked that he flinched if he even saw a hand put near his head, so that this may have been partly hysterical in origin. There was no paralysis of any other cranial nerve than the facial.

### Treatment.

The patient was kept under observation for several days in the hope that some spontaneous improvement might occur with rest and treatment with sedative drugs. So far from this being the case, the weakness of the left arm progressed, the local tenderness became, if possible, even more noticeable, and the pain quite prevented him from sleeping. It was therefore decided to operate, the suggestion being that the condition was either an old depressed fracture with fluid covering it, or an encephalocele.

A large flap of scalp was turned down, the edges of which overlapped the foramen all round by more than an inch. The structures covering the foramen in the skull were then incised and a crescentic flap turned up. Immediately the opening was made clear fluid escaped to the amount of about one to two ounces. The superficial structures appeared to be composed of thickened dura and fibrous tissue, but on completing the flap the cavity which had contained the fluid was found to be the lateral ventricle itself, and a good view was obtained of the choroid plexus lying at the bottom of it. Nothing corresponding to an old depressed fracture was found. The condition seen was difficult to explain, but it must be supposed that the case is one of traumatic encephalocele. The original injury had probably caused a fissured fracture of the vault with damage to the underlying membranes. The fissure, instead of closing, had in course of time become the wide foramen described. This is said sometimes to happen in the course of even a few days or weeks. The portion of cerebral hemisphere lying between the lateral ventricle and the fractured bone had been absorbed and replaced by the layer of thick fibrous tissue which was taken for thickened dura. The membrane lining the ventricle was firmly adherent to the edges of the bone, and no attempt was made to free them. There was no evidence of any inflammatory change in the edges of the bone, a suggestion which had been put forward to account for the extreme tenderness. The bleeding was arrested, the fibrous tissue covering of the lateral ventricle united with catgut, and the wound sewn up. No ill-effects resulted from the operation itself, but this took place too recently for any statement as to the resulting condition of the patient's central nervous system.

